# Successful use of emapalumab in refractory hemophagocytic lymphohistiocytosis in a child with Chédiak–Higashi syndrome: a case report

**DOI:** 10.1186/s13256-023-03808-1

**Published:** 2023-03-29

**Authors:** Ali AlAhmari, Haitham Khogeer

**Affiliations:** 1 Department of Pediatric Hematology/Oncology, King Faisal Specialist Hospital and Research Center, PO Box 3354, Riyadh, 11211 Saudi Arabia; 2 Department of Pathology and Laboratory Medicine, King Faisal Specialist Hospital and Research Center, Riyadh, Saudi Arabia; 3 College of Medicine, AlFaisal University, Riyadh, Saudi Arabia

**Keywords:** Case report, HLH, Chédiak–Higashi syndrome, Emapalumab, HSCT

## Abstract

**Background:**

Hemophagocytic lymphohistiocytosis is a life-threatening disease heralded by fever, cytopenia, hepatosplenomegaly, and multisystem organ failure. Its association with genetic mutations, infections, autoimmune disorders, and malignancies is widely reported.

**Case presentation:**

A 3-year-old male Arab Saudi patient with insignificant past medical history and parental consanguinity presented with abdominal distension of moderate severity and persistent fever despite receiving antibiotics. This was accompanied by hepatosplenomegaly and silvery hair. The clinical and biochemical profiles were suggestive of Chédiak–Higashi syndrome with hemophagocytic lymphohistiocytosis. The patient received the hemophagocytic lymphohistiocytosis-2004 chemotherapy protocol and had multiple hospital admissions mainly due to infections and febrile neutropenia. After achieving the initial remission, the patient’s disease reactivated and did not respond to reinduction with the hemophagocytic lymphohistiocytosis-2004 protocol. Due to the disease reactivation and intolerance of conventional therapy, the patient commenced emapalumab. The patient was successfully salvaged and underwent an uneventful hematopoietic stem cell transplantation.

**Conclusions:**

Novel agents such as emapalumab can be helpful for the management of refractory, recurrent, or progressive disease, while avoiding the toxicities of conventional therapy. Due to a paucity of available data on emapalumab, additional data are needed to establish its role in hemophagocytic lymphohistiocytosis treatment.

## Background

Chédiak–Higashi syndrome (CHS) is a rare autosomal recessive disorder associated with frequent infections and bleeding, oculocutaneous albinism, neurological disorders, and high risk of hemophagocytic lymphohistiocytosis (HLH) [1, 2]. HLH is a hyperinflammatory condition characterizing the “accelerated phase” of CHS and the primary cause of death in CHS patients [2]. The self-perpetuating loop of T cell, natural killer (NK) cell, and macrophage overactivation characteristic of HLH can lead to a cytokine storm, with high mortality if left untreated [3]. HLH can be familial with a clear genetic component (primary HLH), or can be an acquired disorder due to other conditions such as infections and malignancy (secondary HLH) [4]. HLH is primarily complicated by delays in diagnosis and treatment, predominately due to its nonspecific symptoms (fever, cytopenia, hepatosplenomegaly, and multisystem organ failure), and inadequate access to necessary specific laboratory and genetic testing [3, 5].

Allogeneic hematopoietic stem cell transplantation (HSCT) after disease remission is the only potentially curative treatment for both CHS and HLH [6, 7]. The mainstay of HLH treatment remains the combination of immunosuppression, chemotherapy, and biologics targeting the suppression of the cytokine storm and the elimination of overactivated immune cell populations. Coupling dexamethasone and etoposide is a commonly used approach to treat HLH based on the Histiocyte Society HLH-1994 and HLH-2004 recommendations [8]. However, more targeted treatment approaches are essential and are being considered in light of the poor tolerability of conventional therapy and the poor prognosis of relapsed/refractory HLH. Emapalumab is a monoclonal antibody targeted against interferon gamma, with proven efficacy for the treatment of HLH [9], approved for use in both adults and children with relapsed, refractory or progressive disease, or in cases where conventional therapy cannot be used [10]. That being said, experience with emapalumab remains limited and its use is mainly reserved for the second-line setting [11]. As additional data are needed to establish the role of emapalumab in front-line disease management, we present the case of CHS with relapsing HLH, treated with emapalumab prior to successful HSCT. This case could help guide physicians’ decision-making process while treating a challenging case of HLH.

## Case presentation

A 3-year-old Arab Saudi boy was referred to our hospital with a history of recurrent fever which had persisted for ~ 3 months despite receiving antibiotics. The patient had abdominal distension of moderate severity, accompanied by hepatosplenomegaly and silvery hair. Past medical history was insignificant. The patient was born as a full term baby through a normal vaginal delivery without any complications in the neonatal period. Family history was significant for parental consanguinity as the parents are first cousins. Patient has one sibling who is alive and well, with no history of any inherited disorders from the family. There is no history of any allergy and vaccination history is appropriate for patient’s age. Developmentally, all age-appropriate milestones were achieved. Father earns a good income and socioeconomic status is fair.

On arrival to the hospital, the patient had a temperature of 36.2 °C. Other vital signs were normal with a peripheral pulse rate of 109 beats per minute, blood pressure of 107/73 mmHg, respiratory rate of 28 breaths per minute, and oxygen saturation of 100% at room air.

On examination, the patient had an average build with fair skin and silvery hair. Anthropometric measurements were normal with a height of 88 cm, weight of 12 kg, and a body mass index of 16.5 kg/m^2^. Respiratory system examination was normal with bilateral air entry and equal breath sounds. In the cardiovascular system, patient was found to have normal peripheral perfusion and S1 and S2, with no murmur or other abnormality. Patient was neurologically intact with a Glasgow Coma Scale of 15/15, with normal sensory and motor function. No focal defects were detected. In the gastrointestinal examination, abdomen was distended but nontender. There was a massive hepatosplenomegaly with liver 10 cm below the right costal margin and spleen 15 cm below the left costal margin when palpated. Bowel sounds were normal.

His initial laboratory tests at presentation revealed: white blood cells (WBC) 4.39 × 10^9^/L, platelets 23 × 10^9^/L, hemoglobin (Hb) 96 g/L, total bilirubin 21.9 µmol/L, alanine aminotransferase (ALT) 84 U/L, aspartate transaminase (AST) 277 U/L, lactate dehydrogenase (LDH) 459 U/L, prothrombin time (PT) 13.8 seconds, partial thromboplastin time (PTT) 56.3 seconds, triglyceride 6.48 mmol/L, ferritin 894 µg/L, and soluble CD25 5000 pg/mL (for full laboratory results, see Table 1).

**Table 1:** Laboratory findings on admission

Parameter	Status
CBC	
White blood cells	4.39 × 10^9^
Red blood cells	3.92 × 10^12^
Hemoglobin	96 g/L
Hematocrit	0.287 L/L
Platelet	23 × 10^9^/L
Polymorph	7%
Lymphocytes	75%
Monocytes	15%
Liver function test	
Total bilirubin	21.9 µmol/L
Direct bilirubin	4 µmol/L
Lactate dehydrogenase	459 U/L
Alanine aminotransferase	84 U/L
Aspartate aminotransferase	277 U/L
Renal function test	
Blood urea nitrogen	3.3 mmol/L
Creatinine blood	20 µmol/L
Urinalysis	
Glucose	Negative
Protein	Negative
Bilirubin	Negative
Urobilinogen	Negative
pH	7.5
Ketone	Negative
Blood	Negative
Nitrite	Negative
Leukocytes	Negative
Specific gravity	1.005
Serology	
Hepatitis A IgM antibody	Nonreactive
Hepatitis A total antibody	Reactive
Hepatitis B surface antigen	Nonreactive
CMV	Seropositive
EBV	500 IU/mL
Microbiology	
Blood culture	Negative
Fungal culture	Negative
PCR	Negative
Chemistry	
Ferritin	195 µg/L
Fibrinogen	113 mg/dL
Triglyceride	6.48 mmol/L
Prothrombin time	14.7 seconds
Partial thromboplastin time	44.8 seconds
Potassium	3.9 mmol/L
Sodium	137 mmol/L
Blood glucose random	5.6 mmol/L
Hemoglobin electrophoresis	Normal
Coagulation profile	Normal

*CBC* complete blood count, *CMV* cytomegalovirus, *EBV* Epstein–Barr virus, *PCR* polymerase chain reaction

**Fig. 1:**
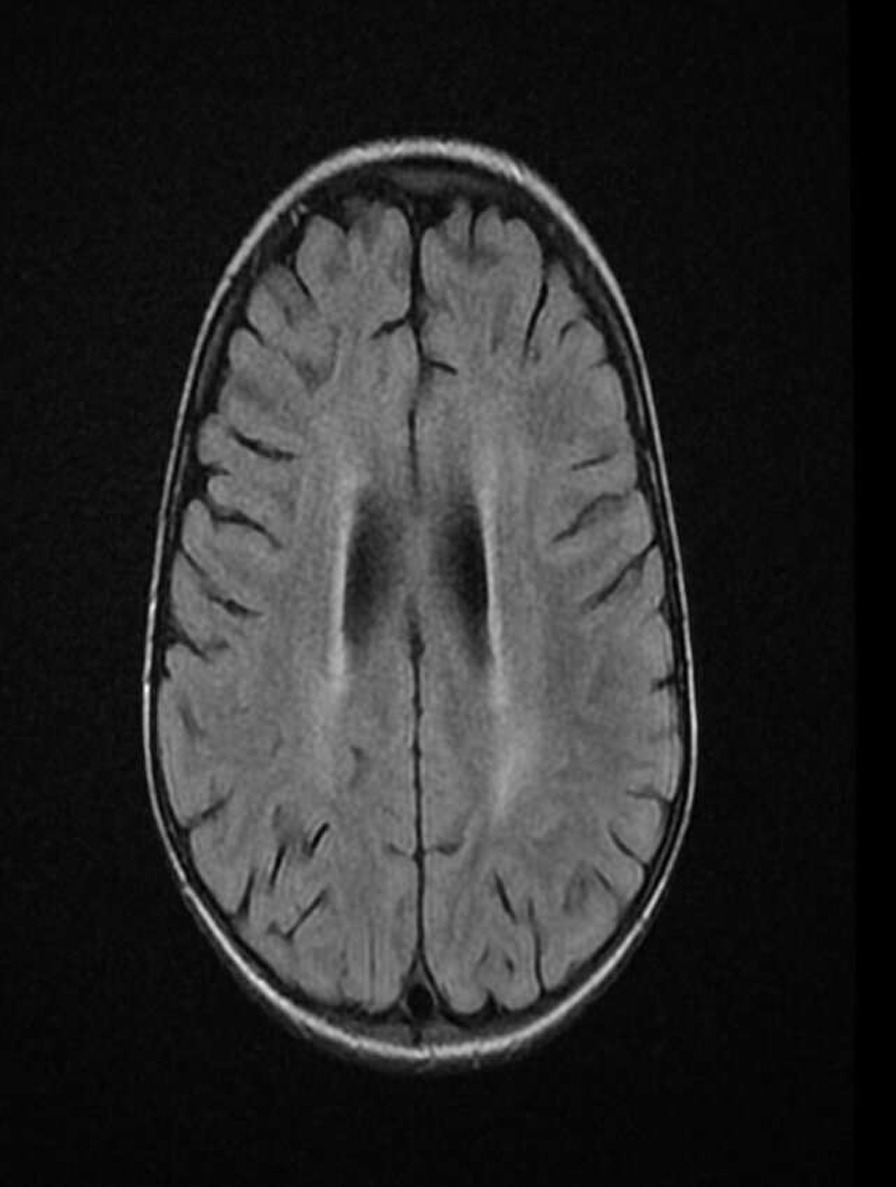
Diffuse brain parenchymal volume loss with cerebral white matter disease

**Fig. 2:**
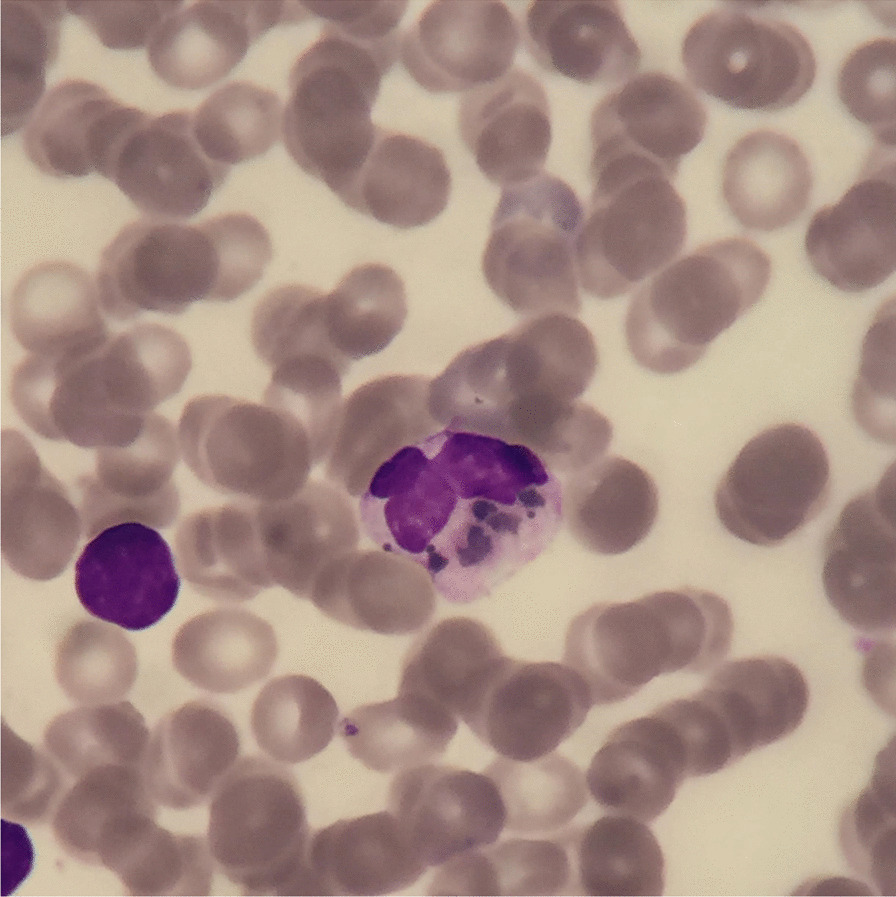
Peripheral blood smear from the patient shows giant granules in the cytoplasm of neutrophils

Hair microscopy revealed hypopigmented hair shafts with pigment clumps, and magnetic resonance imaging findings showed diffuse brain parenchymal volume loss with cerebral white matter disease (Fig. 1). Peripheral blood smear showed giant granules in the cytoplasm neutrophils (Fig. 2). Bone marrow biopsy showed normocellular marrow with 100% cellularity, adequate megakaryocytes, and erythropoiesis. Granulopoieses was adequate with increased lymphocytes and histiocytes. Central nervous system (CNS) involvement, and silvery hair results, were also suggestive of CHS with HLH. Molecular study revealed bi-allelic mutation of the *LYST* gene consistent with CHS.

Treatment history is presented in Table 2. The patient was initially treated on 6 June 2019 with dexamethasone (10 mg/m^2^/day) and intravenous Ig only due to parent refusal of chemotherapy with good initial response resulting in tapering the dose of dexamethasone. However, the patient experienced a disease reactivation that necessitated starting the HLH-2004 on 1 November 2019. Thereafter, the patient experienced multiple disease reactivations associated with febrile neutropenia and infections, requiring several hospital admissions in less than 1 year. On 25 February 2020, the patient was treated with reinduction of HLH-2004 protocol. On 18 April 2020, the patient was admitted for disease reactivation and cytomegalovirus (CMV) infection. The patient was treated again with reinduction of the HLH-2004 chemotherapy protocol and ganciclovir. After achieving the initial remission, patient’s disease reactivated and did not respond to the reinduction with HLH-2004 protocol, with increasing risks of drug toxicities. As a result, the patient received emapalumab from 27 July until 13 August 2020 (six doses, one dose every 3 days) and achieved remission without any significant adverse effects. Reported side effects included erythematous rash over the right forearm, anterior, and posterior thigh after the first dose of emapalumab. There was no further history of rashes, fever, or diarrhea. Treatment with emapalumab was followed by a successful HSCT on 7 September 2020 from a human leukocyte antigen (HLA) identical male sibling. Graft-versus-host disease (GVHD) prophylaxis was given with methotrexate and tacrolimus. Patient received myeloablative conditioning in the form of fludarabine and treosulfan. Pentamidine was used for pneumocystis pneumonia (PCP) prophylaxis. Follow-up at day 100 after transplant showed that the patient recovered the absolute neutrophil count (ANC) on 28 September 2020 (21st day), and platelets were recovered on 1 October 2020 (24th day). Patient developed, hypertension and cytomegalovirus infection, bacterial infection, as well as the fungal infection, which were treated successfully. Granulocyte colony-stimulating factor (GCSF) was used to maintain the ANC > 1000. There was no GVHD, mucositis, veno-occlusive disease, hemorrhagic cystitis, or thrombotic microangiopathic anemia reported during the first 100 days, post-transplant. The patient got engrafted with a picture of mixed chimerism. The patient continued to be in remission and engrafted at 6 months follow-up. GCSF was continued due to persistent neutropenia. Adrenal insufficiency and renal impairment were treated successfully. CMV viremia resolved.

**Table 2:** Routes of administration, duration of treatment, and doses of all medications that were given during hospital stay and follow-up

Medication	Route	Dose	Duration
Dexamethasone	Intravenous	10 mg/m^2^/day	Started on 6 June 2019, stopped on 29 November 2019 Restarted on 9 January 2020, stopped on 4 October 2020
Immunoglobulin	Intravenous	7.5 g	Started on 6 June 2019, stopped on 8 November 2020
FK506 (tacrolimus)	Oral	0.5 mg daily	Started on 3 March 2020, stopped on 30 October 2020
Cyclosporine	Oral	30 mg BID	Started on 26 November 2019, stopped on 2 March 2020
Etoposide	Intravenous	90 mg	Started on 26 October 2019, stopped on 6 May 2020
Emapalumab	Intravenous	50 mg	Started on 27 July 2020, stopped on 13 August 2020
Filgrastim	Intravenous	80 µg	Started on 23 October 2019, stopped 15 December 2019
Ganciclovir	Intravenous	75 mg	Started on 17 March 2020, stopped on 2 June 2020
Conditioning medications			
Treosulfan	Intravenous	10 g daily	Started on day -5 (2 September 2020), stopped on day -3 (4 September 2020)
Fludarabine	Intravenous	30 mg daily	Started on day 6- (1 September 2020), stopped on day -2 (5 September 2020)
**Prophylaxis**			
*GVHD prophylaxis*			
Methotrexate	Intravenous	7.5 mg on days 1, 3, and 6	Started on 8 September 2020, stopped on 13 September 2020
Leucovorin	Intravenous	7.5 mg days 2, 4, and 7	Started on 9 September 2020, stopped on 16 September 2020
FK506 (tacrolimus)	Oral/NGT	0.5 mg daily	Started on day 3 (4 September 2020), stopped on 4 January 2021
*Antiviral prophylaxis*			
Acyclovir	Intravenous	315 mg TID daily	Started on day 3 (4 September 2020), stopped on 9 September 2020
*Antifungal prophylaxis*			
Fluconazole	Oral	100 mg daily	Stared on 17 September 2020, stopped on 30 December 2020
*PCP prophylaxis*			
Pentamidine	Intravenous	70 mg (once every 4 weeks	Started on day 3 (4 September 2020), stopped on 5 July 2021
**Others**			
Ursodiol	Oral	150 mg, BID	Started on 30 August 2020, stopped on 30 December 2020

*BID* twice daily, *GVHD* graft-versus-host-disease, *PCP* pneumocystis pneumonia, *TID* three times daily

The patient continued to have a mixed chimerism at 6 months. Evolution of lymphoid and myeloid donor cells chimerism was as follows (respectively): 82% and 76% on 2 November 2020, 82% and 47% on 30 November 2020, 88% and 40% on 4 January 2021, 86% and 27% on 1 February 2021.

## Discussion and conclusions

The presented case is that of a 3-year-old Arab Saudi male patient presenting with clinical evidence suggestive of Chédiak–Higashi syndrome with HLH. The patient had refractory recurrent disease; disease reactivation occurred after the first remission, with failure to respond to reinduction with the HLH-2004 protocol. Due to disease reactivations and intolerance of conventional therapy, the patient commenced emapalumab. The patient was successfully salvaged and underwent uneventful hematopoietic stem cell transplant. This case illustrates that alternative treatment options, such as emapalumab can lead to remission in a challenging clinical scenario of frequently relapsing disease, with no major toxicity, thus allowing the conduction of the only potentially curative treatment for HLH and Chédiak–Higashi syndrome.

HLH is a very common clinical manifestation of primary immunodeficiencies, such as CHS, and a major driver of mortality rates in young patients [12, 13].

Genetic mutations are implicated in both CHS and HLH. Consanguinity rates remain high in Saudi Arabia, where familial history of HLH is also relatively common [14]. Establishing the genetic profile of HLH and the prevalent mutations in local populations is important to improve early diagnosis and treatment. *STXBP2* and *STX11* gene mutations were previously reported to be the two most predominant mutations in Saudi patients with HLH [15]. Our findings fit the spectrum of pathogenic variants reported for nonfamilial HLH in Saudi Arabia, where pathogenic variants of the *LYST* gene (some of which were novel mutations) were detected in 5.8% of all mutant patients [15]. These novel homozygous *LYST* gene mutations consequently confirmed the diagnosis of CHS [15].

In addition to CNS involvement, concurrent infections (viral or bacterial infections) seem to be intimately related to HLH [12, 13, 16]. Viral infections such as severe acute respiratory syndrome coronavirus 2 (SARS-CoV-2), Epstein–Barr virus (EBV), and CMV have been reported to trigger HLH in infants and children, with or without primary immunodeficiencies [17, 18]. Several bacterial (*Klebsiella*), fungal (*Candida*), and viral (EBV, CMV) infections were observed over the course of the presented patient’s management. CMV infection was evident upon disease reactivation in the presented case, prompting the use of the HLH-2004 protocol along with ganciclovir. One study suggests that disease remission could be more likely in infection-associated HLH, while EBV-related disease leads to a higher risk of relapse [19]. Long-term outcomes of the HLH-2004 study showed improved 5-year survival rates compared with the HLH-94 protocol. The HLH-2004 protocol actually ensured up to 71% 5-year survival in children without family history or genetically verified disease [20].

That being said, the use of the HLH-2004 protocol does not always yield the desired clinical response, with patients experiencing partial response, disease recurrence, and death [19, 21, 22]. Our patient ultimately did not respond to reinduction with the HLH-2004 protocol. Alternative options were therefore needed to achieve disease remission and bridge to HSCT, particularly considering the increasing toxicity risk of previous therapy.

It is known that patients with HLH still experience major toxicities and unfavorable outcomes remain common despite major advances in treatment modalities. As previously mentioned, allogeneic HSCT after disease remission is the only potentially curative treatment for both CHS and HLH [6, 7]. However, access to HSCT could be limited in developing countries, with few patients undergoing transplantation [23]. Previous experience from King Faisal Specialist Hospital showed 40% survival after full match HSCT of Saudi CHS patients [24]. In a cohort of Saudi patients with HLH, HSCT greatly improved 5-year overall survival rates to reach at least 66.5% of patients [15]. Conditioning regimens could play an important role in preventing graft failure and improving clinical outcomes and survival after HSCT [25]. Early reports of the use of etoposide in local CHS patients undergoing HSCT at King Faisal Specialist Hospital were positive in terms of management of the accelerated phase, but not conditioning before HSCT [26].

Globally, emapalumab is increasingly being considered as bridge therapy to curative HSCT, and as a way to improve survival and clinical outcomes after transplantation [27]. This was reflected in our case, where treatment with emapalumab led to remission in a frequently relapsing disease with no major toxicity, thus allowing a successful HSCT. Available evidence supports the use of emapalumab for the treatment of severe refractory HLH, due to its efficacy, safety, and tolerability, and the absence of effect on control of concurrent viral and other infections [9, 28, 29]. The use of emapalumab has been suggested for EBV-related HLH to reduce the risk of secondary malignancy due to etoposide [18]. Regardless, clinicians should be aware of the potential drawbacks of inducing severe immunosuppression, and the consequent inhibition of the febrile response to infection, with the use of emapalumab in combination with other immunomodulatory agents such as dexamethasone and etoposide [30].

Despite our center being one of the biggest HLH centers in the world, various challenges are still faced in the treatment of HLH, particularly patients with refractory disease and disease reactivation. In this case, emapalumab proved to be effective in achieving disease remission, without any major complications, in less than a month. It also decreased the patient’s readmission requirements and hospital length of stay, all of which were of major concern in this case. That being said, one limitation of this case could be the absence of any substantial data to support the use of emapalumab at our center, which in turn emphasizes the need for more studies to endorse emapalumab use in local HLH populations.

Even with the advancements made in HLH treatment, significant unfavorable outcomes and toxicities remain common. Treatment-refractory and relapsing disease remains especially challenging. The presented case provides support to the notion that novel agents such as emapalumab can be helpful for the management of refractory, recurrent, or progressive disease, and clinical scenarios where treatment options become limited by the toxicities of conventional therapy. While emapalumab seems to be promising in the management of challenging HLH cases, more insights and robust studies are needed to explore its role in first-line disease treatment.

## Data Availability

Not applicable.

## Abbreviations

Ab: Antibody
ALT: Alanine aminotransferase
AST: Aspartate transaminase
BM: Bone marrow
CHS: Chédiak–Higashi syndrome
CMV: Cytomegalovirus
CNS: Central nervous system
CSA: Cyclosporin A
D100: Day 100
EBV: Epstein–Barr virus
FK506: Tacrolimus
GCSF: Granulocyte colony-stimulating factor
GVHD: Graft-versus-host disease
Hb: Hemoglobin
HLA: Human leukocyte antigen
HLH: Hemophagocytic lymphohistiocytosis
HSC: Hematopoietic stem cell
HSCT: Hematopoietic stem cell transplantation
IVIG: Intravenous immune globulin
LDH: Lactate dehydrogenase
MTX: Methotrexate
PT: Prothrombin time
PTT: Partial thromboplastin time
TNC: Total nucleated cell
VOD: Veno-occlusive disease
VP16: Etoposide
WBC: White blood cell
